# Photocatalytic Activity of Cu_2_S/WO_3_ and Cu_2_S/SnO_2_ Heterostructures for Indoor Air Treatment

**DOI:** 10.3390/ma14133656

**Published:** 2021-06-30

**Authors:** Alexandru Enesca, Luminita Isac

**Affiliations:** 1 Product Design, Mechatronics and Environmental Department, Transilvania University of Brasov, Eroilor 29 Street, 35000 Brasov, Romania; 2 Renewable Energy Systems and Recycling Research Center, Product Design, Mechatronics and Environmental Department, Transilvania University of Brasov, Eroilor 29 Street, 35000 Brasov, Romania; isac.luminita@unitbv.ro

**Keywords:** photocatalysis, semiconductors, heterostructure, S-scheme mechanism, acetaldehyde, air decontamination

## Abstract

Volatile organic compounds (VOCs) are commonly found in indoor spaces (e.g., homes or offices) and are often related to various illnesses, some of them with carcinogenic potential. The origins of VOC release in the indoor environment are in office products, building materials, electronics, cleaning products, furniture, and maintenance products. VOC removal can be done based on two types of technologies: adsorption in specific materials and decomposition via oxidative processes. The present article reports the development and photocatalytic activity of two heterostructures (Cu_2_S/WO_3_ and Cu_2_S/SnO_2_) used for indoor air decontamination. The acetaldehyde removal rate is discussed in correlation with the S-scheme mechanisms established between the heterostructure components but also comparatively with the bare catalysts’ activity. Acetaldehyde was considered as a VOC reference because it was found by the International Agency for Research on Cancer to be one of the most frequent air toxins with potential carcinogenic effects. The samples contained monoclinic WO_3_, tetragonal SnO_2_, and orthorhombic Cu_2_S crystalline structures. The Cu_2_S crystallite size in the heterostructure varied from 75.9 to 82.4 Å, depending on the metal oxide substrate. The highest photocatalytic efficiency (75.7%) corresponded to Cu_2_S/SnO_2_, with a constant rate of 0.106 s^−1^ (which was three times faster than WO_3_ or SnO_2_ and seven and a half times faster than Cu_2_S).

## 1. Introduction

The development of urban areas and industrial activities has significantly decreased indoor and outdoor air quality. Volatile organic compounds (VOC) are commonly found in indoor spaces (home or office) and are often related to various illnesses, some of them with carcinogenic potential [1,2,3]. The origins of VOC release in the indoor environment are in office products, building materials, electronics, cleaning products, furniture and maintenance products. VOC removal can be done based on two types of technologies: adsorption in specific materials and decomposition via oxidative processes [4,5,6]. The adsorption technique is able to capture the pollutant molecules into the sorbent media in a stable form because of the formation of chemical or physical bindings. This method is commonly used in office spaces but has as disadvantages the fast sorbent saturation and removal capability [7,8]. Additionally, the increase in air flow rate or temperature will induce VOC desorption and environmental re-contamination. After saturation, the sorbent must be carefully treated and disposed in special containers, considering the contaminant’s hazard potential [9,10].

The decomposition processes use oxidation techniques: photocatalysis, ozonation, or plasma-driven oxidation [11,12,13]. The photocatalytic air decontamination has the advantage of being activated by the light energy reaching the catalyst surface. Consequently, the process can be designed to be energy sustainable because of the use of sunlight or low-energy-consuming light sources. In this case, the catalyst must be a material with high receptivity to both UV and vis spectra. The catalysts are represented by a single component, tandems/heterostructures, or composite materials, which can be activated by the photon absorption with energy equal or higher than the band-gap value [14,15,16]. Single catalysts such as TiO_2_ [17], WO_3_ [18], ZnO [19], SnO_2_ [20], and CuInS_2_ [21] show important limitations in terms of reduced light absorbance, fast electron-hole recombination, or low chemical stability in the working environment. Composite materials (graphene/ZnO, textiles/TiO_2_, fly ash/semiconductors, etc.) combine both photocatalytic and absorbance processes and usually exhibit high removal efficiencies [22,23,24]. The main issue is related with the incomplete decomposition of the absorbed pollutant, which has the potential to be released in the environment. Finally, the heterostructures such as TiO_2_/CuInS_2_ (CIS) [25], TiO_2_/WO_3_ [26], SnO_2_/ZnO [27], or ZnO/WO_3_ [28] have the advantage of using an extended light spectra, a reduced charge recombination, and the ability to generate a high quantity of oxidative radicals.

Using innovative materials such as cellulose particles [29], graphene-based nanomaterials and zeolite [30] with high VOC adsorbent capacity represents another approach for gas pollutant removal. These types of adsorbents can reduce their capacity when they reach the saturation point or the adsorption equilibrium. The photocatalytic oxidation of VOCs is a difficult process, and the decomposition pathways and efficiencies depend on various reaction conditions. The humidity is a very important factor influencing VOCs’ degradation [31]. High concentrations of water vapor in the working environment are beneficial to the photocatalytic oxidation of VOCs because of the increase in hydroxyl radical development, acting as an oxidizing agent on the surfaces of the photocatalytic materials [32]. However, water molecules and VOC molecules can compete for filling the adsorption active sites, which reduce the photocatalytic efficiency [33].

The present article reports the development and photocatalytic activity of two heterostructures (Cu_2_S/WO_3_ and Cu_2_S/SnO_2_) used for indoor air decontamination. The acetaldehyde removal rate is discussed in correlation with the Z-scheme mechanisms established between the heterostructure components but also comparatively with bare photocatalyst activity. The influence of the crystalline structure, morphology, and surface composition was also investigated. The results indicated that a facile chemical route procedure can be employed to obtain efficient photocatalytic heterostructures able to work in the removal of toxic pollutants.

## 2. Materials and Methods

### 2.1. Heterostructure Development

Three mono-component and two heterostructures were obtained by the sol-gel technique as follows:

(i) WO_3_ powder was prepared by adding tungsten hexachloride (WCl_6_, 99.8%, Acros Organics, Geel, Belgium) in a solvent composed of 2-propanol (100%, Sigma Aldrich, Munich, Germany) and ethanol (100%, Sigma Aldrich, Munich, Germany). The above composition was stirred for 140 min until a light yellow solution was formed. The gel formed after the slow addition of 0.21 mol natrium hydroxide (99.98%, Honeywell, Charlotte, NC, USA). The precipitate was centrifuged, and the resulting powder was thermally treated for 7 h at 450 °C.

(ii) SnO_2_ powder was obtained by dissolving tin tetrachloride (SnCl_4_, 99.7%, Sigma Aldrich, Munich, Germany) in absolute ethanol (100%, Sigma Aldrich, Munich, Germany). A white solution was obtained after 120 min of stirring at 40 °C. Then, the 0.18 mol of sodium hydroxide (99.98%, Honeywell, Charlotte, NC, USA) was added drop by drop until the gel was formed. The resulting precipitate was centrifuged and the powder was annealed for 5 h at 500 °C.

(iii) Cu_2_S powder was obtained from an aqueous solution containing 0.3 mol of copper nitrate (Cu(NO_3_)_2_, 99.9%, Scharlau, Barcelona, Spain) and 0.5 mol of sodium thiosulfate (Na_2_S_2_O_3_, 99.9%, Scharlau, Barcelona, Spain). The gel was formed after 30 min of stirring and kept in the dark for 6 h to achieve the complete precipitation. After centrifugation, the powder containing intermediary compounds based on CuS_2_O_3_ and Cu_2_S_2_O_3_ was thermally treated at 140 °C in a ceramic capsule containing a sulfured (sulfur, 99%, Sigma Aldrich, Munich, Germany) atmosphere. The ceramic capsule was inserted into a furnace (Nabertherm, Lilienthal, Germany) containing an Ar atmosphere (Linde, Dubline, Ireland). The low-oxygen-content atmosphere during the thermal treatment is a pre-requisite to ensure the Cu_2_S stoichiometry.

(iv) The Cu_2_S/WO_3_ powder heterostructure was prepared based on the same procedure as described for Cu_2_S. The WO_3_ powder was inserted into a copper nitrate precursor considering the Cu:W atomic ratio of 1:1. The sample was annealed at 140 °C for 2 h.

(v) The Cu_2_S/SnO_2_ powder heterostructure was obtained by adding the SnO_2_ powder into the copper nitrate solution, and the stirring period was increased up to 1 h. The Cu:Sn atomic ratio was 1:1, and the final powder was thermally treated for 3 h at 150 °C.

### 2.2. Photocatalytic Experiments

The photocatalytic activity of the mono-component and the heterostructures was tested into a cylindrical quartz air-proof photoreactor (Sigma Aldrich, Munich, Germany). Firstly, the photoreactor was filled with dry air using a continuous flow rate for 30 min. Secondly, the photoreactor containing 0.05 g of catalyst was injected with 150 ppm acetaldehyde (Linde, Dubline, Ireland). The mixture was kept in the dark for 2 h to reach the absorption equilibrium. Finally, the quartz photoreactor was uniformly irradiated with a UV source (0.08 mW/cm^2^, 280–400 nm) for 12 h, in order to ensure that photons were evenly distributed on the catalyst surface. The changes in acetaldehyde and carbon dioxide concentrations were recorded by gas chromatography (GC-2014, Shimadzu, Maryland, CO, USA).

### 2.3. Characterization

The formation of the crystalline structure was identified by X-ray diffraction (XRD) analysis made with a Rigaku Miniflex X-Ray diffractometer (Tokyo, Japan) using a Cu K_α_ source (I = 1.54 Å, 40 kV, 100 mA). The mono-components’ and heterostructures’ morphology was investigated by field emission scanning electron microscopy (FESEM, SU8010, Fukuoka, Japan) with an accelerated voltage of 25 kV and high-vacuum scanning electron microscopy (SEM, Hitachi model S–3400 N type 121 II, Tokyo, Japan). Both devices were able to investigate the surface elemental composition of the samples. The irradiation source was tested with a class-A high-precision pyranometer (SR11, Hukselflux, Berlin, Germany), and the acetaldehyde degradation was evaluated with gas chromatography (GC-2014, Shimadzu, Maryland, CO, USA).

## 3. Results and Discussions

### 3.1. Composition and Morphology

**Figure 1:**
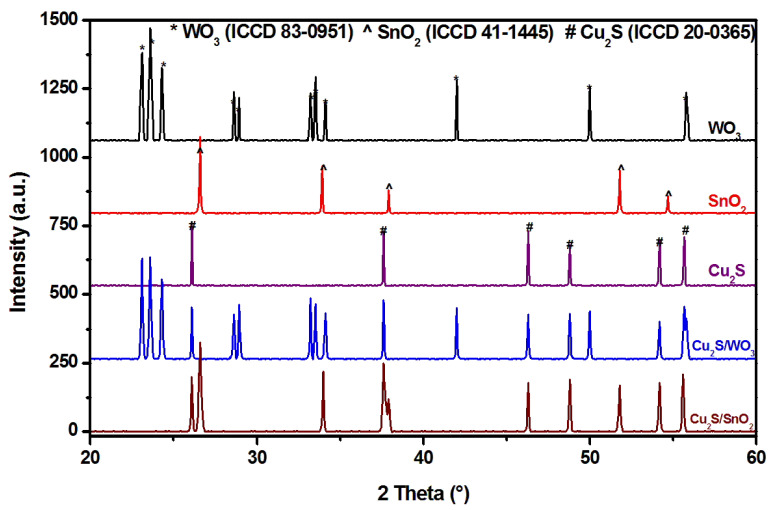
X-ray diffraction (XRD) patterns of the mono-component and heterostructure samples.

The diffraction investigations are presented in Figure 1. Table 1 contains the average crystallites size evaluated based on each XRD samples pattern. The results indicate that all mono-component samples contained the crystalline structure corresponding to the designated semiconductor. Monoclinic WO_3_ (ICCD 83-0951), tetragonal SnO_2_ (ICCD 41-1445), and orthorhombic Cu_2_S (ICCD 20-0365) were formed after the power thermal treatments followed the particular parameters presented in Section 2. The metal oxides presented higher crystallite sizes (93.8 Å for WO_3_ and 81.5 Å for SnO_2_) comparatively with Cu_2_S (64.7 Å), where milder thermal conditions were used to avoid sulfur sublimation [34,35,36]. The benchmark samples preserved the stoichiometry of the envisaged compounds that would constitute the basis of heterostructure catalysts and the substrate for Cu_2_S development. The heterostructures both contained the crystalline structure of the specific components, but the Cu_2_S crystallite size was influenced by the addition of the metal oxide into the synthesis precursor. Consequently, the Cu_2_S developed a higher crystallite size in the presence of WO_3_, where the space limitation restriction was lower than that of SnO_2_. These results are similar with other reports [37,38,39] indicating the Cu_2_S will use the highest energy sites, which were, in this case, the metal oxide particle, as preferential nucleation points. The WO_3_ exhibited the larger crystallite sizes, which favor the Cu_2_S growth mechanism and the formation of extensive component interfaces. The SnO_2_ induced space limitation but had no negative influence on the nucleation process. Even if there were no indications on the formation of other components because of ion diffusion, this process cannot be excluded considering that they may have been in an amorphous state [40,41,42].

**Figure 2:**
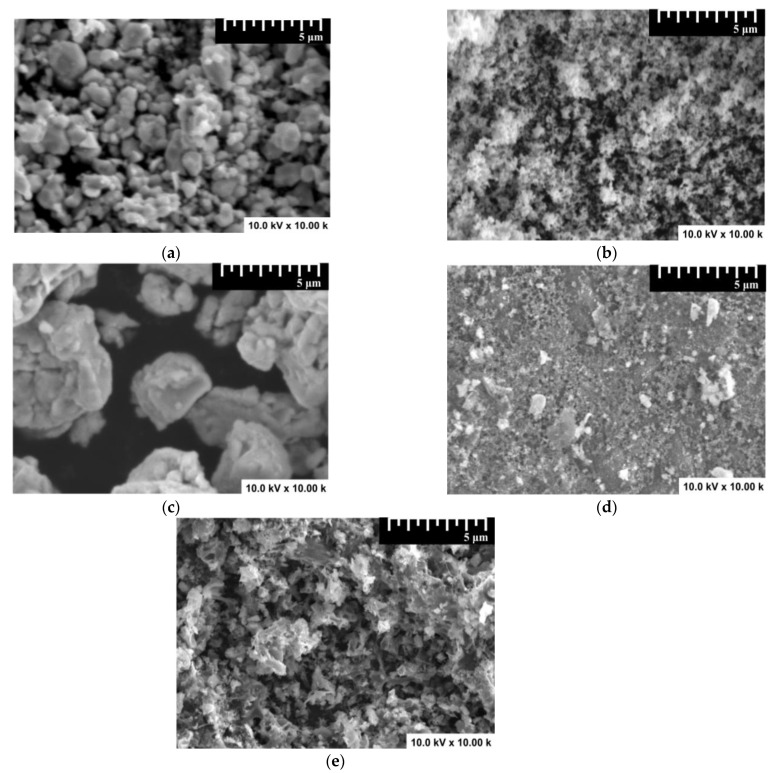
Scanning electron microscopy (SEM) images of the photocatalysts: (**a**) WO_3_, (**b**) SnO_2_, (**c**) Cu_2_S, (**d**) Cu_2_S/WO_3_, and (**e**) Cu_2_S/SnO_2_.

The SEM analysis presented in Figure 2 indicates that the mono-component samples’ morphology was severely influenced by the sol-gel synthesis parameters. The metal oxides exhibited dispersed particles, because of the use of an alcoholic precursor, inducing the gel formation during the sodium hydroxide slow addition. The high annealing temperature and NaOH concentration favored the formation of WO_3_ particles with sizes varying from 0.5 to 2 µm. The use of a lower NaOH concentration and a shorter annealing treatment would induce the formation of small SnO_2_ particles with an average size of 200 nm. The aqueous precursor employed for Cu_2_S synthesis allowed the formation of large aggregates composed of particles with sizes varying from 0.5 to 3 µm. As described by Beneto et al. [43], the heterostructures’ morphology is influenced by the insertion of metal oxide particles, which serve as a Cu_2_S-growing substrate. Cu_2_S/WO_3_ showed a dense morphology containing particles with various shape and sizes combined in a relatively compact assembly. The Cu_2_S development was done on small and uniformly distributed SnO_2_, allowing the formation of a closely connected network with large cavities that can be used as photoactive sites during the acetaldehyde decomposition [44,45]. The metal oxides had a higher Brunauer-Emmett-Teller (BET) surface area (36.8 m^2^/g for WO_3_ and 44.2 m^2^/g for SnO_2_) compared with the copper sulfide (21.4 m^2^/g) component. The heterostructures used the metal oxides as substrates for Cu_2_S development, which would influence the overall active surface during the photocatalytic activity. Consequently, the Cu_2_S/SnO_2_ exhibited a larger BET surface area (41.5 m^2^/g) than that of Cu_2_S/WO_3_ (32.6 m^2^/g).

The elemental composition evaluation was done based on energy-dispersive X-ray spectroscopy (EDS) measurements undertaken during the morphology investigations, and the results are presented in Table 2. The investigation was influenced by the bulk composition as the penetration index could not be limited to the surface layer. The samples’ photocatalytic activity depended mostly on the homogenous surface composition, considering that the acetaldehyde removal is an interface-dependent process [46,47]. The results were compared with the theoretical values calculated based on the compounds’ stoichiometry identified by the diffraction analysis. The metal oxides’ mono-component samples exhibited oxygen excess due to the oxygen atmosphere used for the annealing treatment. However, the Cu_2_S benchmark showed a sulfur deficit, which was consistent with other studies [48,49,50] indicating the tendency of sulfur sublimation during the thermal treatment. The sublimation was significantly reduced by the low thermal temperature treatment and Ar environment around the capsule. Additionally, the presence of a small amount of oxygen in the Cu_2_S sample indicates the possible formation of amorphous copper oxide, which was not found by XRD measurements. The heterostructure analysis indicated that the atomic ratio between the metals was preserved as presented in the synthesis method. However, a deficit of oxygen and sulfur was identified in both heterostructure compositions, which was due to the metal oxide’s partial coverage with the Cu_2_S component. Defect formations, such as vacancies or interstices, were highly expected based on the EDS results and can influence the overall photocatalytic activity.

### 3.2. Photocatalytic Activity and Mechanism

**Figure 3:**
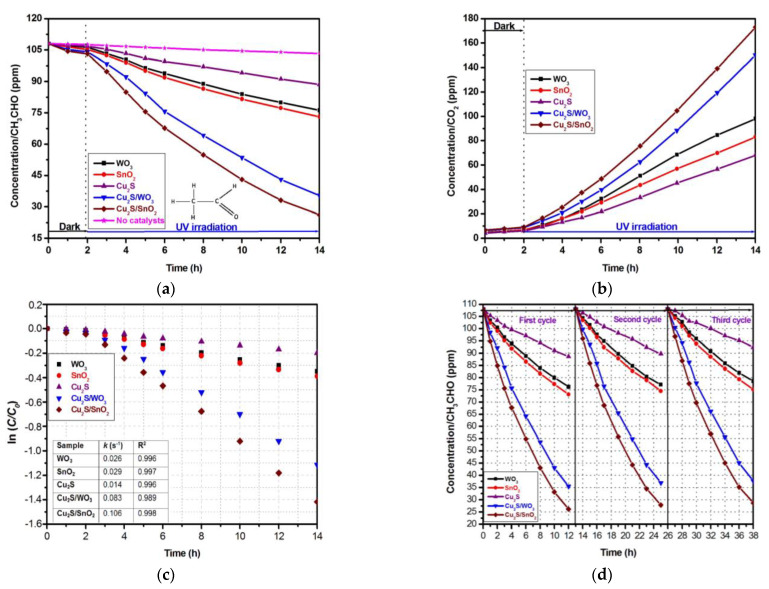
Photocatalytic activity of the mono-component and heterostructure samples: (**a**) acetaldehyde removal, (**b**) CO_2_ formation, (**c**) kinetic evaluation, and (**d**) long stability tests.

The photocatalytic activity was performed using a hermetic quartz photoreactor and a catalyst dosage of 0.05 g for 150 ppm acetaldehyde. The acetaldehyde and CO_2_ concentration evolutions during the experiments were evaluated and are presented in Figure 3a,b. The differences between CH_3_CHO removal and CO_2_ production can be explained by the by-product formations (such as C_2_H_2_O_4_, CH_2_O, etc.), which were not quantified during the experiments. The samples were uniformly irradiated from all sides, using an UV irradiance of 0.08 mW/cm^2^. The absorption equilibrium was reached after keeping the samples in the dark for 2 h. In the next 12 h, the samples were irradiated and evaluated hourly, in the first 4 h, and at 2 h intervals for the remaining period. Acetaldehyde was considered as a VOC reference because it was found by the International Agency for Research on Cancer to be one of the most frequent indoor air toxins with potential carcinogenic effects [51,52]. The photocatalytic acetaldehyde removal efficiency was calculated considering the initial (*C*_0_) and final (*C*) concentrations based on Equation (1):

(1) $$\eta =\left[\frac{\left(C_{0}-C\right)}{C_{0}}\right]\times 100$$

The mono-component samples exhibited low photocatalytic properties with a maximum 32.3% acetaldehyde removal efficiency for SnO_2_ and a minimum acetaldehyde removal efficiency of 17.9% for Cu_2_S. The WO_3_ sample exhibited a 22% acetaldehyde removal efficiency after 12 h of irradiation and the photocatalytic activity remain stable after 3 cycles. In the same photocatalytic testing condition, TiO_2_ showed a maximum acetaldehyde removal efficiency of 15% [53], which is similar with bare Cu_2_S. Another study [54] showed that using composite sheets of titanium dioxide (TiO_2_) and an adsorbent nylon film with a 0.5–5.0% mass ratio is possible to remove 250 ppm acetaldehyde after 240–300 min irradiation with UV (80 μW/cm^2^). Titania can be doped with Fe using chemical vapor deposition and thermal treatment at 750 °C [55]. The photocatalytic experiments were done at different relative humidities (0%, 30%, and 60%), and the results indicated that the acetaldehyde removal efficiency was facilitated by the water vapor’s content. The complete oxidation of acetaldehyde was gradually enhanced with increasing humidity. Commercial TiO_2_ was combined with TaS_2_ to study the photocatalytic degradation of gaseous acetaldehyde [56]. The second component, TaS_2_, provided two merits: (i) a higher adsorptive capacity of gaseous acetaldehyde compared with TiO_2_ and (ii) a better separation efficiency of charge carriers. The system was able to remove 98% of acetaldehyde using vis light as an irradiation source.

The photocatalytic activity increased significantly when heterostructures were involved. The maximum photocatalytic efficiency was obtained for Cu_2_S/SnO_2_ (75.7%), with lamellar morphology allowing cavity formation and a multi-scattering effect. The Cu_2_S/SnO_2_ heterostructure reached 67.2% acetaldehyde removal efficiency, which is consistently higher than that of bare catalysts. The kinetic evaluation presented in Figure 3c was done based on the Langmuir–Hinshelwood model, which considers the concentration, the time (*t*), and the constant rate (*k*) in the following equation:

(2) $$\ln C=\ln C_{0}-kt$$

The results indicate a superior constant rate corresponding to the heterostructures’ photocatalytic activity, compared with mono-component samples. The Cu_2_S/SnO_2_ exhibited an acetaldehyde degradation rate three times higher than that of WO_3_ or SnO_2_ and seven and a half times higher than that of Cu_2_S. The increase in the photocatalytic performance by developing active heterostructure may be the key for fast VOC removal from indoor spaces. The long-term stability tests presented in Figure 3b indicate small changes of the photocatalytic properties after three cycles, excepting the Cu_2_S sample, which showed a significant reduction in the photocatalytic activity between the second and third cycle. However, when coupled with metal oxides in heterostructures, the Cu_2_S exhibited good stability as the exposed liquid-catalysts interface was composed of metal oxides and sulfides.

**Figure 4:**
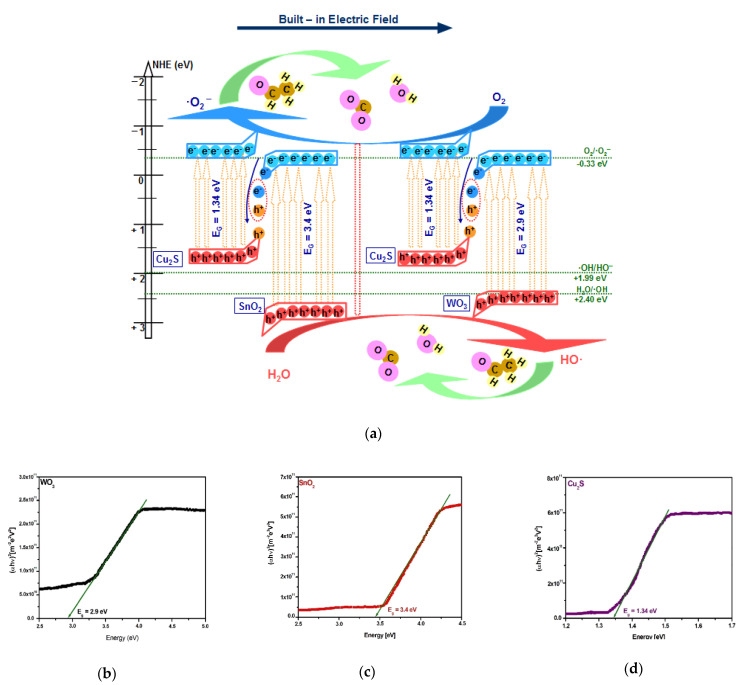
(**a**) Heterostructures’ Z-scheme mechanism and band-gap values of (**b**) WO_3_, (**c**) SnO_2_, and (**d**) Cu_2_S semiconductors.

The photocatalytic activity enhancement corresponding to the Cu_2_S/SnO_2_ and Cu_2_S/WO_3_ heterostructures was elucidated by studding the mechanism behind the pollutant mineralization. The production of oxidative species (HO·, ·O_2_^−^) required charge carriers’ transition and migration, which was based on the photon energy conversion during the irradiation. The development of a band energy diagram consisted in evaluating the experimental band-gap values of each component forming the heterostructure and is presented in Figure 4b–d. The methodology is in good agreement with the literature [57,58], considering that the band-gap heterostructures values shift because of the internal energy field. The evaluation of the energy band position, presented in Figure 4a, includes the integration of several parameters such as the free electron energy vs. hydrogen (*E_e_*), the absolute cationic electronegativity (*χ_cation_*), the semiconductor electronegativity (*χ_semiconductor_*), and the specific cationic electronegativity *χ_cation_* (*P.u.*), where *P.u*. represents the Pauling units and band gap energy (*E_g_*), into Equations (3)–(6).

(3) $$E_{VB}=\chi_{semiconductor}-E_{e}+0.5E_{g}$$

(4) $$E_{CB}=E_{VB}-E_{g}$$

(5) $$\chi_{semiconductor}(eV)=0.45\times \chi_{cation}(eV)+3.36$$

(6) $$\chi_{cation}(eV)=\frac{\chi_{cation}(P.u)+0.206}{0.336}$$

During the light irradiation, the photogenerated electrons originating from the Cu_2_S conduction band (−0.41 eV) would be transferred to SnO_2_ (+0.54 eV) or WO_3_ (+0.52 eV) conduction bands. Owing to their potential, the photoinduced electrons from SnO_2_ and WO_3_ conduction bands (CB) cannot produce ·O_2_^−^, and the photoinduced holes from the Cu_2_S valence band (+0.93 eV) cannot be involved in ·OH generation. These charge carriers cannot be used in the acetaldehyde photocatalytic degradation and will recombine [59,60,61]. The useful photogenerated electrons from the Cu_2_S conduction band (−0.41 eV), and the photogenerated holes from SnO_2_ (+3.94 eV)/WO_3_ (+3.42 eV) valence bands, possessed a stronger redox ability. Consequently, these charge carriers can be efficiently separated by the electric field formed in the charged space region. The synergy between the combined drift and diffusion effect would promote the transfer of photogenerated charge carriers through the heterostructure semiconductor components [62,63,64,65]. This behavior was attributed to the Z-scheme mechanism able to efficiently convert the photon energy in order to produce the charge carriers involved in the development of (super)oxidative species. The Cu_2_S/SnO_2_ heterostructure benefits from the higher SnO_2_ conduction band potential required for charge separation and generation of ·OH radicals. Additionally, the Cu_2_S/SnO_2_ had a higher BET surface area, which increased the interfacial contact with the pollutant gas where the acetaldehyde decomposition takes place. 

## 4. Conclusions

A simple sol-gel procedure was employed to produce three mono-components (WO_3_, SnO_2_, and Cu_2_S) and two heterostructure (Cu_2_S/WO_3_ and Cu_2_S/SnO_2_) photocatalysts. The metal oxides were used as nucleation sites for Cu_2_S development during the synthesis. The samples contained monoclinic WO_3_, tetragonal SnO_2_, and orthorhombic Cu_2_S crystalline structures. The Cu_2_S crystallite size in the heterostructures varied from 75.9 to 82.4 Å, depending on the metal oxide substrate. The Cu_2_S/WO_3_ morphology was characterized by a compact assembly of particles with various sizes and shapes. The surface elemental composition indicated that both heterostructures exhibited an oxygen and sulfur deficit, compared with the stoichiometric composition.

The photocatalytic removal of acetaldehyde was done using a 0.08 mW/cm^2^ UV irradiance. The heterostructures had a significantly higher photocatalytic activity compared with the bare samples. The Cu_2_S/SnO_2_ and Cu_2_S/WO_3_ followed a Z-scheme mechanism allowing the efficient use of charge carriers with a stronger redox ability. The highest photocatalytic efficiency (75.7%) corresponded to Cu_2_S/SnO_2_, with a constant rate of 0.106 s^−1^ (which was three times faster than WO_3_ or SnO_2_ and seven and a half times faster than Cu_2_S). The use of the Z-scheme-mechanism heterostructures can improve the photon energy conversion and reduce the useful charge carrier recombination. The results indicate that the heterostructures containing Cu_2_S have a better stability of the photocatalytic activity compared with the bare Cu_2_S. The main issues were represented by the necessity to increase the photocatalytic efficiency in the presence of a wider light spectrum (UV and vis). Future work will investigate the optimization of heterostructure composition for sunlight applications.

## Figures and Tables

**Table 1:** Photocatalysts’ crystallite size values evaluated based on the Scherrer formula.

Photocatalyst	Crystallite Size (Å)		
	Cu_2_S	WO_3_	SnO_2_
WO_3_	-	93.8	-
SnO_2_	-	-	81.5
Cu_2_S	64.7	-	-
Cu_2_S/WO_3_	82.4	92.6	-
Cu_2_S/SnO_2_	75.9	-	82.6

**Table 2:** Average surface atomic composition of the photocatalysts by energy-dispersive X-ray spectroscopy (EDS).

Sample	Elemental Composition (% at)						
	Cu	Sn	W	O	Oth ^1^	S	Sth ^1^
WO_3_	-	-	23.2	76.8	69.6	-	-
SnO_2_	-	31.4	-	68.6	62.8	-	-
Cu_2_S	71.5	-	-	3.7	-	24.8	35.7
Cu_2_S/WO_3_	17.7	-	16.8	57.6	50.4	7.9	8.8
Cu_2_S/SnO_2_	24.8	-	23.1	41.3	46.2	10.8	12.4

^1^ Theoretic content calculated based on the stoichiometry.

## Data Availability

Data presented in this study are available by requesting from the corresponding author.
